# Duodenal duplication: a rare cause of abdominal pain in adults

**DOI:** 10.11604/pamj.2019.33.303.19779

**Published:** 2019-08-19

**Authors:** Soumaya Mrabet, Mohamed Salah Jarrar, Amine Gouader, Imen Akkari, Elhem Ben Jazia

**Affiliations:** 1 Department of Gastroenterology, Farhat Hached University Hospital, Sousse, Tunisia; 2 Department of Surgery, Farhat Hached University Hospital, Sousse, Tunisia

**Keywords:** Abdominal pain, adult, duodenal duplication, surgery

## Abstract

Duodenal duplication is an extremely rare congenital abnormality that occurs mostly in children. It represents only 2% to 12% of all gastrointestinal tract duplication. Its clinical presentation is highly variable and non-specific making the positive diagnosis very difficult. Imaging modalities can help to detect the lesions making the diagnosis more accurate before surgery. Here, we report a case of duodenal duplication revealed by chronic abdominal pain and treated by surgical resection in a 26-year-old man. Even in adults, it is necessary to evoke the diagnosis of duodenal duplication in patients with unexplained abdominal pain. Surgical resection remains the treatment of choice and endoscopic treatment is reserved for selected patients in whom surgery is difficult.

## Introduction

Duodenal duplication (DD) is a rare congenital malformation that occurs oftenly in early childhood, but could also be discovered at any period of life. The diagnosis remains difficult and can be challenging [1]. The management of DD is often surgical. Endoscopic resection has also been reported as an alternative choice [2]. This report describes our experience with DD in an adult and reviews clinical implications and treatment.

## Patient and observation

**Figure 1:**
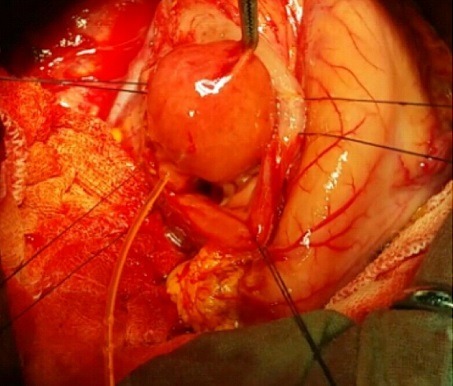
Intraoperative view: a three cm spherical structure adjoined to the duodenal wall just near the biliopancreatic ampulla

**Figure 2:**
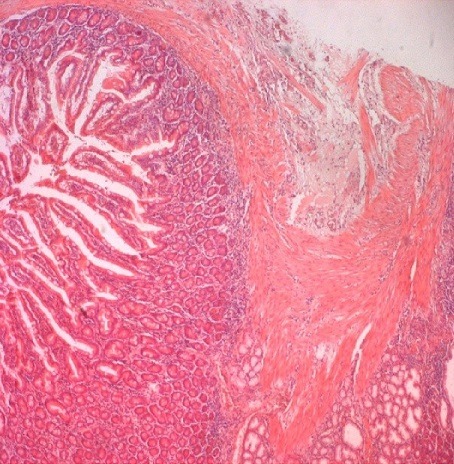
Histological finding from the resected cyst revealed duodenum like mucosa that displayed a distinct layer of smooth muscle and macked villi

A 26-year-old man presented with recurrent epigastric pain evolving since three months associated with intermittent jaundice and fever. There was no history of vomiting, anorexia or change in bowel habits. Physical examination was normal and laboratory values were normal. A Computed tomography (CT) of the abdomen revealed the presence of 3.5×3 cm cystic mass within the second portion of the duodenum near the head of the pancreas without pancreaticobiliairy ductal dilatation. Endoscopic ultrasonography showed a double layered wall of the second part of the duodenum with a cystic lesion. Magnetic resonance cholangiopancreatography (MRCP) revealed that the cyst was confined to the duodenum and did not involve the intra-pancreatic portion of the common bile duct. These appearances are consistent with the diagnosis of a DD. The patient underwent an open abdominal exploration by laparotomy. A limited duodenotomy was performed on the anterior surface of the second portion of the duodenum. We found three-cm spherical structure adjoined to the duodenal wall just near the biliopancreatic ampulla (Figure 1). These findings were again suggestive of a duodenal duplication. The duodenal duplication was fully mobilized and totally resected. The postoperative course was uneventful. Histological findings from the resected cyst revealed duodenum like mucosa that displayed a distinct layer of smooth muscle and macked villi structure without signs of malignancy (Figure 2).

## Discussion

Fitz gave the first description of a DD in 1884 [3]. DD in adults represents only 2% to 12% of all alimentary tract duplication. Indeed, the duplications of gastrointestinal tract occur most commonly in the distal ileum, followed by the esophagus, colon and jejunum [4]. Jo *et al* defined three criteria for the diagnosis, including the presence of an intimate attachment to the native gastrointestinal tract, a muscle coat and alimentary mucosal lining [5]. DD can be divided into tubular or cystic, communicating or non-communicating. Most are located in the first or the second portions of the duodenum [6]. Clinical manifestations are often non-specific. In adults, the major symptom is abdominal pain. Other signs are vomiting, jaundice, abdominal distension and gastrointestinal bleeding can occur due to the presence of ectopic gastric mucosa [2]. Imaging modalities can help detect the lesions to make the diagnosis become more accurate. The goal of endoscopic ultrasonography is to reveal the relationship of the DD and the pancreaticobiliary system, which can differentiate this lesion from the choledochal cyst, especially Todani type III [1]. The surgical intervention for duodenal duplication cyst includes complete or partial surgical resection of the cyst depending on the relation to ampulla [4]. Recently, some reports are suggesting the endoscopic management of these cysts in adults with good long-term outcomes [7]. It has been reported that DD has the possibility to become malignant [8]. Therefore, surgical resection remains the treatment of choice and endoscopic treatment is reserved for selected patients in whom surgery is difficult. In our case, we treated our patient with total excision which cured the patient.

## Conclusion

DD is a relatively rare congenital anomaly that may occur anywhere along the gastro-intestinal tract. It is necessary to evoke this diagnosis in front of unexplained abdominal pain. Endoscopic approach is possible but requires operative endoscopic skills.

## Competing interests

The authors declare no competing interests.
